# Crystal structure of *N*-(1*H*-indol-2-yl­methyl­idene)-4-meth­oxy­aniline

**DOI:** 10.1107/S2056989022002973

**Published:** 2022-03-31

**Authors:** Masatsugu Taneda, Masato Nishi, Koji Kubono, Yukiyasu Kashiwagi, Taisuke Matsumoto

**Affiliations:** a Department of Science Education, Faculty of Education, Osaka Kyoiku University, Kashiwara, Osaka 582-8582, Japan; b Division of Natural Sciences, Osaka Kyoiku University, Kashiwara, Osaka 582-8582, Japan; c Osaka Research Institute of Industrial Science and Technology, 1-6-50 Morinomiya, Joto-ku, Osaka 536-8553, Japan; d Institute for Materials Chemistry and Engineering, Kyushu University, Kasuga, Fukuoka 816-8580, Japan

**Keywords:** crystal structure, indole, Schiff base, bidentate ligand, C—H⋯π inter­actions

## Abstract

The mol­ecule of the title compound, C_16_H_14_N_2_O, contains an essentially planar indole ring system and a phenyl ring. In the crystal, the mol­ecules are linked by a weak inter­molecular C—H⋯O hydrogen bond and C—H⋯π inter­actions, forming a one-dimensional column structure along the *b-*axis direction. These columns are linked by other C—H⋯π inter­actions, forming a two-dimensional network structure.

## Abstract 2

The mol­ecule of the title compound contains an essentially planar indole ring system and a phenyl ring. In the crystal, the mol­ecules are linked by a weak inter­molecular C—H⋯O hydrogen bond and C—H⋯π inter­actions, forming a two-dimensional network structure.

## Chemical context

Indole and its derivatives are useful starting compounds to derive pharmaceutical (Nalli *et al.*, 2020 ▸) and biological materials (Arumugam *et al.*, 2021 ▸). Indole can function as a hydrogen-bond donor because of the high acidity of the hydrogen atom at position 1. The introduction of a hydrogen-bond acceptor to position 2 of the indole ring forms a five-to-seven-membered intra­molecular hydrogen-bonded ring (Nosenko, *et al.*, 2008 ▸). In this work, a Schiff base including an indole ring, *N*-(indol-2-yl­methyl­idene)-4-meth­oxy­aniline, was newly synthesized. Similar Schiff bases such as salicyl­idene­amines often function as bidentate ligands (Wang *et al.*, 2018 ▸). Whereas salicyl­idene­amines form intra­molecular hydrogen bonds between coordination site atoms, such intra­molecular inter­actions are absent from the crystal structure of the title compound. We report herein on its mol­ecular and crystal structure. 
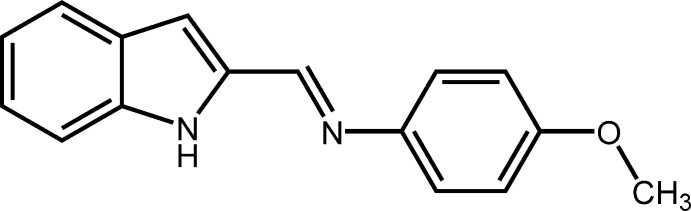


## Structural commentary

**Figure 1:**
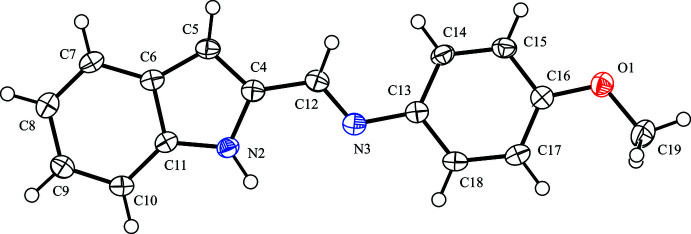
The mol­ecular structure of the title compound with atom labelling. Displacement ellipsoids are drawn at the 50% probability level. H atoms are represented by spheres of arbitrary radius.

The mol­ecular structure of the title compound is shown in Fig. 1 ▸. The C=N double bond adopts an *E* configuration. The indole moiety is almost planar with an r.m.s. deviation of 0.009 (1) Å. The bond lengths and angles in the title mol­ecule are normal and agree with those in other indole imine compounds (IWIGUS; Suresh *et al.*, 2016 ▸; KEVLON; Ho *et al.*, 2006 ▸). The dihedral angle between the indole system and the benzene ring is 9.89 (5)°. In the related compound IWIGUS, the dihedral angles between the indole system and the benzene ring disordered over two sets of sites are widened to 81.8 (3) and 85.2 (3)° due to two isopropyl substituents in the benzene ring. There is no intra­molecular hydrogen bond in the title compound, because the N2—H2⋯N3 angle is as small as 94.4 (10)°; however, the N2⋯N3 distance is 2.8633 (16) Å, and the N2—C4—C12—N3 torsion angle is 3.94 (19)°. Although no intra­molecular hydrogen bond is observed, a broad peak assigned for the N—H proton is seen in the ^1^H NMR spectrum of the title compound in a CDCl_3_ medium and this suggests that the compound forms an intra­molecular hydrogen bond in solution (see *Synthesis and crystallization*).

## Supra­molecular features

**Figure 2:**
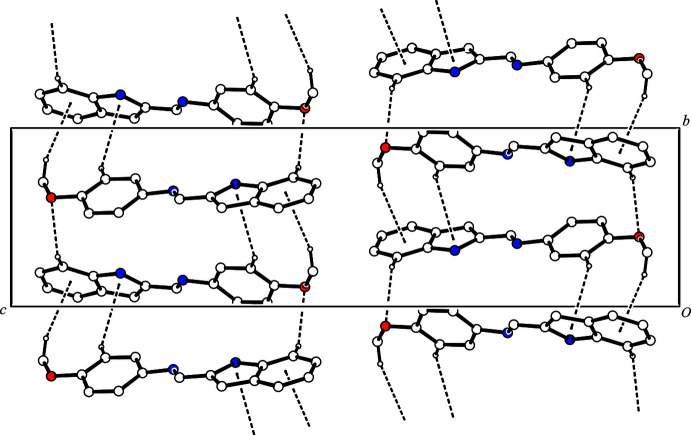
One-dimensional column structure in the crystal of the title compound viewed along the *a* axis. The C—H⋯O hydrogen bonds and the C—H⋯π inter­actions are shown as dashed lines. H atoms not involved in these inter­actions are omitted for clarity.

**Figure 3:**
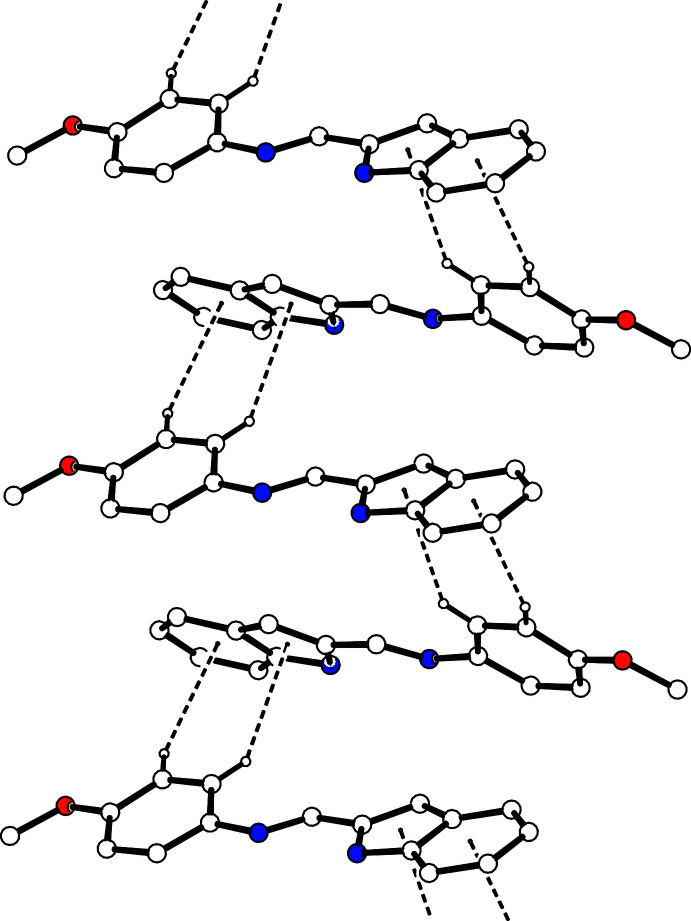
Part of the crystal structure of the title compound showing the formation of ribbons along the *b*-axis direction. The C—H⋯π inter­actions are shown as dashed lines. H atoms not involved in these inter­actions are omitted for clarity.

**Figure 4:**
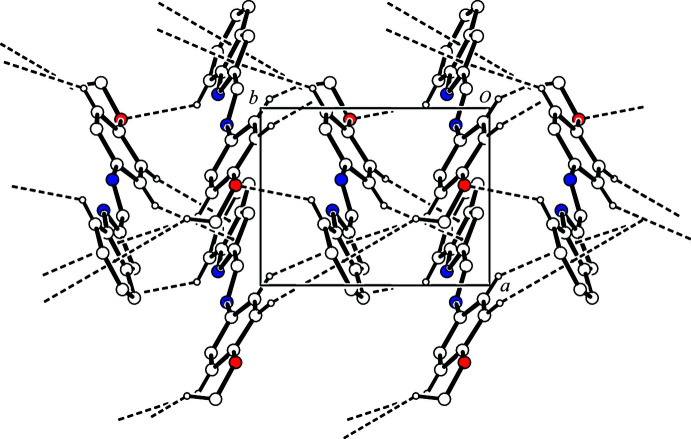
A packing diagram of the title compound viewed along the *c* axis, showing the two-dimensional network. The C—H⋯O hydrogen bonds and C—H⋯π inter­actions are shown as dashed lines. H atoms not involved in these inter­actions are omitted for clarity.

The title compound contains an N—H group, which is a hydrogen-bond donor, and an imino group, which is a hydrogen-bond acceptor, but neither of them forms an inter­molecular hydrogen bond in the crystal. Compounds containing a similar indol-2-yl­methyl­idene-aniline fragment with a *cis*-conformation of the C—C single bond between the N atoms often form dimers by inter­molecular N—H⋯N hydrogen bonds (see *Database survey*). However, in the crystal the mol­ecules of the title compound are linked by a weak inter­molecular C10—H10⋯O1^i^ hydrogen bond and C—H⋯π inter­actions [C17—H17⋯*Cg*1^i^ and C19—H19*C*⋯*Cg*2^i^; *Cg*1 is the centroid of the N2/C4–C6/C11 ring and *Cg*2 is the centroid of the C6–C11 ring; symmetry code: (i) 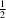 − *x*, 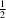 + *y*, 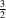 − *z*], forming columns along the *b-*axis direction (Fig. 2 ▸, Table 1 ▸). Besides this, the mol­ecules belonging to different columns are joined by other C—H⋯π inter­actions [C14—H14⋯*Cg*1^ii^ and C15—H15⋯*Cg*2^ii^; symmetry code: (ii) 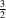 − *x*, − 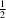 + *y*, 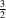 − *z*] (Fig. 3 ▸, Table 1 ▸). As a result, the inter­molecular C—H⋯O hydrogen bonds and C—H⋯π inter­actions form a two-dimensional network structure (Fig. 4 ▸).

## Database survey

A search of the Cambridge Structural Database (CSD, Version 5.42, update of May 2021; Groom *et al.*, 2016 ▸) using *ConQuest* (Bruno *et al.*, 2002 ▸) for indole derivatives gave 5272 hits, and for the (1*H*-indol-2-yl)methanimine skeleton gave 86 hits. Among these, the imino N atom bonded to an H atom gave one hit, to an N atom gave 24 hits, and to a C atom gave 61 hits. A search for the indol-2-yl­methyl­idene-aniline fragment gave 30 hits, and those containing a (1*H*-indol-2-yl)methyl­idene-aniline fragment with a *cis*-conformation of the C—C single bond gave seven hits. These seven compounds include five examples of dimers linked by complementary N—H⋯N hydrogen bonds (FORJAA; Li *et al.*, 2019 ▸; IWIGUS; Suresh *et al.*, 2016 ▸; KEZCUQ; Ariyasu *et al.*, 2016 ▸; VACKES; Gadekar *et al.*, 2016 ▸; WAGCEP; Tian *et al.*, 2016 ▸), one example of a one-dimensional-chain structure (UWUSAI; Kalalbandi & Seetharamappa, 2016 ▸), and one example of a monomer protected from hydrogen bonding by steric hindrance (KEVLON; Ho *et al.*, 2006 ▸). These structures contain inter­molecular or intra­molecular hydrogen bonds involving the N—H or the imino groups. Of these structures, the compounds most closely related to the title compound are *N*-(2,6-diiso­propyl­phen­yl)-1-(1*H*-indol-2-yl)methanimine (IWIGUS; Suresh *et al.*, 2016 ▸), 4,6-dimeth­oxy-3-methyl-2,7-bis­[(phenyl­imino)­meth­yl]indole (KEVLON; Ho *et al.*, 2006 ▸) and 2-(phenyl-*N*-oxido­imino­meth­yl)-3-phenyl­amino­indole (CIP­WED; Greci & Sgarabotto, 1984 ▸). In the crystal of IWIGUS, which features a large dihedral angle between the indole and benzene rings, two neighbouring mol­ecules are associated through pairs of N—H⋯N inter­molecular hydrogen bonds, forming a centrosymmetric dimer. The crystal structure of an indol-2-yl­methyl­idene-aniline compound without a hydrogen bond between the N—H and imino groups has not yet been reported. In an almost planar mol­ecule without a bulky substituent such as the tile compound, the formation of a dimer by inter­molecular N—H⋯N hydrogen bonding is probably not appropriate for the crystal packing.

## Synthesis and crystallization

Indole-2-carbaldehyde (145 mg, 1.00 mmol) and *p*-anisidine (148 mg, 1.20 mmol) were dissolved in toluene (20 mL), and the solution was refluxed under inert gas for 6 h, followed by evaporation. The residue was purified by recrystallization from a solvent mixture of acetone and *n*-hexane (1:1), and the title compound was then obtained (212 mg, 0.848 mmol, 84.8%) as a pale-red powder. The recrystallization of the title compound from a mixture of acetone and methanol afforded single crystals suitable for X-ray structure analysis. ^1^H NMR (CDCl_3_, 400 MHz) *δ* = 3.84 (*s*, 3H, OCH_3_), 6.93–6.97 (*m*, 3H, ArH), 7.13 (*td*, 1H, *J_ortho_* = 7.5 Hz, *J_meta_* = 1.0 Hz, ArH), 7.25–7.31 (*m*, 3H, ArH), 7.40 (*dd*, 1H, *J_ortho_* = 8.3 Hz, *J_meta_* = 0.9 Hz, ArH), 7.66 (*d*, 1H, *J_ortho_* = 8.0 Hz, ArH), 8.48 (*s*, 1H, N=CH), 9.25 (*br*, 1H, NH). HR–MS (*m*/*z*): calculated for [C_16_H_15_N_2O_]^+^, *m*/*z* = 251.1179; found, 251.1192.

## Refinement

Crystal data, data collection and structure refinement details are summarized in Table 2 ▸. The H atom attached to N2 was located in a difference-Fourier map and freely refined. The C-bound H atoms were positioned geometrically and refined using a riding model: C—H = 0.93–0.96 Å with *U*_iso_(H) = 1.2*U*_eq_(C).

## Supplementary Material

Crystal structure: contains datablock(s) global, I. DOI: 10.1107/S2056989022002973/yk2166sup1.cif

Structure factors: contains datablock(s) I. DOI: 10.1107/S2056989022002973/yk2166Isup2.hkl

Supporting information file. DOI: 10.1107/S2056989022002973/yk2166Isup3.cml

CCDC reference: 2159620

Additional supporting information:  crystallographic information; 3D view; checkCIF report

## Figures and Tables

**Table 1:** Hydrogen-bond geometry (Å, °) *Cg*1 and *Cg*2 are the centroids of the N2/C4–C6/C11 and C6–C11 rings, respectively.

*D*—H⋯*A*	*D*—H	H⋯*A*	*D*⋯*A*	*D*—H⋯*A*
C10—H10⋯O1^i^	0.93	2.58	3.2479 (16)	129
C14—H14⋯*Cg*1^ii^	0.93	2.81	3.6006 (14)	143
C15—H15⋯*Cg*2^ii^	0.93	2.79	3.5153 (14)	136
C17—H17⋯*Cg*1^i^	0.93	2.89	3.5718 (14)	131
C19—H19*C*⋯*Cg*2^i^	0.96	2.97	3.7716 (16)	142

Symmetry codes: (i) 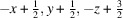 ; (ii) 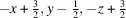 .

**Table 2:** Experimental details

Crystal data	
Chemical formula	C_16_H_14_N_2_O
*M*_r_	250.29
Crystal system, space group	Monoclinic, *P*2_1_/*n*
Temperature (K)	123
*a*, *b*, *c* (Å)	5.87685 (19), 7.5999 (3), 28.4578 (11)
β (°)	90.604 (3)
*V* (Å^3^)	1270.95 (8)
*Z*	4
Radiation type	Mo *K*α
μ (mm^−1^)	0.08
Crystal size (mm)	0.30 × 0.20 × 0.10

Data collection	
Diffractometer	Rigaku AFC10 Saturn70 area detector
Absorption correction	Multi-scan *CrysAlis PRO*; Rigaku OD, 2018 ▸)
*T*_min_, *T*_max_	0.608, 0.992
No. of measured, independent and observed [*F* ^2^ > 2.0σ(*F* ^2^)] reflections	11128, 2907, 2525
*R*_int_	0.056
(sin θ/λ)_max_ (Å^−1^)	0.649

Refinement	
*R*[*F* ^2^ > 2σ(*F* ^2^)], *wR*(*F* ^2^), *S*	0.049, 0.127, 1.05
No. of reflections	2907
No. of parameters	178
H-atom treatment	H atoms treated by a mixture of independent and constrained refinement
Δρ_max_, Δρ_min_ (e Å^−3^)	0.39, −0.29

Computer programs: *CrysAlis PRO* (Rigaku OD, 2018 ▸), *SIR92* (Altomare *et al.*, 1993 ▸), *SHELXL2018/3* (Sheldrick, 2015 ▸), *PLATON* (Spek, 2020 ▸) and *CrystalStructure* (Rigaku, 2019 ▸).

## supplementary crystallographic information

### Crystal data

**Table:**

C_16_H_14_N_2_O	*F*(000) = 528.00
*M**_r_* = 250.29	*D*_x_ = 1.308 Mg m^−^^3^
Monoclinic, *P*2_1_/*n*	Mo *K*α radiation, λ = 0.71073 Å
*a* = 5.87685 (19) Å	Cell parameters from 6329 reflections
*b* = 7.5999 (3) Å	θ = 2.8–30.8°
*c* = 28.4578 (11) Å	µ = 0.08 mm^−^^1^
β = 90.604 (3)°	*T* = 123 K
*V* = 1270.95 (8) Å^3^	Prism, colourless
*Z* = 4	0.30 × 0.20 × 0.10 mm

### Data collection

**Table:**

Rigaku AFC10 Saturn70 area detector diffractometer	2907 independent reflections
Radiation source: rotating anode X-ray generator, micromax007	2525 reflections with *F*^2^ > 2.0σ(*F*^2^)
Multi-layer mirror optics monochromator	*R*_int_ = 0.056
Detector resolution: 28.5714 pixels mm^-1^	θ_max_ = 27.5°, θ_min_ = 2.8°
ω scans	*h* = −7→7
Absorption correction: multi-scan CrysAlisPro; Rigaku OD, 2018)	*k* = −8→9
*T*_min_ = 0.608, *T*_max_ = 0.992	*l* = −36→35
11128 measured reflections	

### Refinement

**Table:**

Refinement on *F*^2^	Hydrogen site location: inferred from neighbouring sites
*R*[*F*^2^ > 2σ(*F*^2^)] = 0.049	H atoms treated by a mixture of independent and constrained refinement
*wR*(*F*^2^) = 0.127	*w* = 1/[σ^2^(*F*_o_^2^) + (0.0686*P*)^2^ + 0.3253*P*] where *P* = (*F*_o_^2^ + 2*F*_c_^2^)/3
*S* = 1.05	(Δ/σ)_max_ = 0.001
2907 reflections	Δρ_max_ = 0.39 e Å^−^^3^
178 parameters	Δρ_min_ = −0.29 e Å^−^^3^
0 restraints	Extinction correction: SHELXL
Primary atom site location: structure-invariant direct methods	Extinction coefficient: 0.259 (14)
Secondary atom site location: difference Fourier map	

### Special details

**Table:**

Geometry. All esds (except the esd in the dihedral angle between two l.s. planes) are estimated using the full covariance matrix. The cell esds are taken into account individually in the estimation of esds in distances, angles and torsion angles; correlations between esds in cell parameters are only used when they are defined by crystal symmetry. An approximate (isotropic) treatment of cell esds is used for estimating esds involving l.s. planes.
Refinement. Refinement was performed using all reflections. The weighted R-factor (wR) and goodness of fit (S) are based on F^2^. R-factor (gt) are based on F. The threshold expression of F^2^ > 2.0 sigma(F^2^) is used only for calculating R-factor (gt).

### Fractional atomic coordinates and isotropic or equivalent isotropic displacement parameters (Å^2^)

**Table:**

	*x*	*y*	*z*	*U*_iso_*/*U*_eq_	
O1	0.06455 (16)	0.60985 (13)	0.93970 (3)	0.0292 (3)	
N2	0.57779 (18)	0.68608 (14)	0.66418 (4)	0.0237 (3)	
N3	0.40410 (18)	0.64748 (14)	0.75724 (4)	0.0248 (3)	
C4	0.7005 (2)	0.61479 (16)	0.70151 (4)	0.0240 (3)	
C5	0.9058 (2)	0.55340 (17)	0.68542 (5)	0.0249 (3)	
H5	1.0199	0.5007	0.7034	0.030*	
C6	0.9115 (2)	0.58536 (16)	0.63609 (4)	0.0221 (3)	
C7	1.0717 (2)	0.55168 (17)	0.60070 (5)	0.0253 (3)	
H7	1.2079	0.4950	0.6079	0.030*	
C8	1.0237 (2)	0.60388 (17)	0.55530 (5)	0.0272 (3)	
H8	1.1287	0.5822	0.5318	0.033*	
C9	0.8174 (2)	0.68975 (17)	0.54398 (5)	0.0266 (3)	
H9	0.7899	0.7250	0.5131	0.032*	
C10	0.6548 (2)	0.72278 (17)	0.57776 (4)	0.0253 (3)	
H10	0.5182	0.7780	0.5701	0.030*	
C11	0.7037 (2)	0.67011 (16)	0.62387 (4)	0.0219 (3)	
C12	0.6095 (2)	0.60207 (17)	0.74822 (4)	0.0245 (3)	
H12	0.7019	0.5599	0.7724	0.029*	
C13	0.3193 (2)	0.62794 (16)	0.80344 (4)	0.0231 (3)	
C14	0.4210 (2)	0.52631 (17)	0.83908 (5)	0.0264 (3)	
H14	0.5511	0.4612	0.8327	0.032*	
C15	0.3296 (2)	0.52215 (17)	0.88355 (5)	0.0266 (3)	
H15	0.3993	0.4548	0.9069	0.032*	
C16	0.1332 (2)	0.61828 (16)	0.89388 (4)	0.0233 (3)	
C17	0.0233 (2)	0.71292 (17)	0.85845 (5)	0.0253 (3)	
H17	−0.1111	0.7729	0.8645	0.030*	
C18	0.1179 (2)	0.71653 (17)	0.81370 (5)	0.0247 (3)	
H18	0.0446	0.7798	0.7900	0.030*	
C19	−0.1427 (3)	0.6969 (2)	0.95149 (5)	0.0367 (4)	
H19A	−0.1793	0.6722	0.9836	0.044*	
H19B	−0.2632	0.6554	0.9313	0.044*	
H19C	−0.1247	0.8215	0.9474	0.044*	
H2	0.438 (3)	0.737 (2)	0.6678 (5)	0.032 (4)*	

### Atomic displacement parameters (Å^2^)

**Table:**

	*U*^11^	*U*^22^	*U*^33^	*U*^12^	*U*^13^	*U*^23^
O1	0.0294 (5)	0.0304 (5)	0.0279 (5)	0.0010 (4)	0.0024 (4)	−0.0028 (4)
N2	0.0196 (5)	0.0258 (6)	0.0258 (5)	0.0019 (4)	−0.0007 (4)	−0.0004 (4)
N3	0.0257 (5)	0.0218 (6)	0.0270 (6)	−0.0006 (4)	−0.0012 (4)	0.0001 (4)
C4	0.0223 (6)	0.0226 (6)	0.0270 (7)	−0.0032 (5)	−0.0028 (5)	−0.0007 (5)
C5	0.0216 (6)	0.0242 (6)	0.0288 (7)	−0.0008 (5)	−0.0046 (5)	0.0014 (5)
C6	0.0194 (6)	0.0180 (6)	0.0289 (7)	−0.0027 (4)	−0.0030 (5)	−0.0015 (5)
C7	0.0192 (6)	0.0228 (6)	0.0339 (7)	0.0008 (5)	−0.0006 (5)	−0.0029 (5)
C8	0.0247 (6)	0.0259 (7)	0.0310 (7)	−0.0034 (5)	0.0029 (5)	−0.0056 (5)
C9	0.0289 (6)	0.0249 (7)	0.0258 (6)	−0.0022 (5)	−0.0032 (5)	−0.0005 (5)
C10	0.0235 (6)	0.0238 (6)	0.0283 (7)	0.0021 (5)	−0.0040 (5)	0.0000 (5)
C11	0.0194 (6)	0.0189 (6)	0.0273 (6)	−0.0015 (5)	−0.0009 (4)	−0.0023 (5)
C12	0.0237 (6)	0.0239 (7)	0.0259 (6)	−0.0032 (5)	−0.0042 (5)	−0.0007 (5)
C13	0.0221 (6)	0.0200 (6)	0.0270 (6)	−0.0023 (5)	−0.0017 (5)	−0.0005 (5)
C14	0.0216 (6)	0.0242 (7)	0.0335 (7)	0.0047 (5)	0.0029 (5)	0.0042 (5)
C15	0.0250 (6)	0.0243 (7)	0.0305 (7)	0.0019 (5)	−0.0022 (5)	0.0053 (5)
C16	0.0239 (6)	0.0192 (6)	0.0266 (6)	−0.0043 (5)	−0.0009 (5)	−0.0025 (5)
C17	0.0206 (6)	0.0212 (6)	0.0340 (7)	0.0012 (5)	−0.0017 (5)	−0.0023 (5)
C18	0.0227 (6)	0.0222 (6)	0.0289 (7)	0.0009 (5)	−0.0054 (5)	0.0016 (5)
C19	0.0341 (8)	0.0398 (9)	0.0364 (8)	0.0040 (6)	0.0069 (6)	−0.0064 (7)

### Geometric parameters (Å, º)

**Table:**

O1—C16	1.3705 (15)	C9—H9	0.9300
O1—C19	1.4287 (17)	C10—C11	1.3989 (17)
N2—C11	1.3769 (16)	C10—H10	0.9300
N2—C4	1.3876 (16)	C12—H12	0.9300
N2—H2	0.912 (17)	C13—C18	1.3950 (17)
N3—C12	1.2840 (17)	C13—C14	1.4035 (18)
N3—C13	1.4189 (17)	C14—C15	1.3803 (18)
C4—C5	1.3768 (18)	C14—H14	0.9300
C4—C12	1.4413 (18)	C15—C16	1.4000 (18)
C5—C6	1.4252 (18)	C15—H15	0.9300
C5—H5	0.9300	C16—C17	1.3918 (18)
C6—C7	1.4094 (18)	C17—C18	1.3950 (18)
C6—C11	1.4205 (17)	C17—H17	0.9300
C7—C8	1.3781 (19)	C18—H18	0.9300
C7—H7	0.9300	C19—H19A	0.9600
C8—C9	1.4111 (18)	C19—H19B	0.9600
C8—H8	0.9300	C19—H19C	0.9600
C9—C10	1.3852 (19)		
			
C16—O1—C19	117.50 (11)	C10—C11—C6	121.79 (12)
C11—N2—C4	108.89 (10)	N3—C12—C4	121.67 (12)
C11—N2—H2	128.4 (10)	N3—C12—H12	119.2
C4—N2—H2	122.7 (10)	C4—C12—H12	119.2
C12—N3—C13	119.83 (11)	C18—C13—C14	118.05 (12)
C5—C4—N2	109.15 (11)	C18—C13—N3	116.78 (11)
C5—C4—C12	128.24 (12)	C14—C13—N3	125.17 (12)
N2—C4—C12	122.51 (11)	C15—C14—C13	120.54 (12)
C4—C5—C6	107.42 (11)	C15—C14—H14	119.7
C4—C5—H5	126.3	C13—C14—H14	119.7
C6—C5—H5	126.3	C14—C15—C16	120.68 (12)
C7—C6—C11	119.10 (12)	C14—C15—H15	119.7
C7—C6—C5	134.07 (12)	C16—C15—H15	119.7
C11—C6—C5	106.83 (11)	O1—C16—C17	125.06 (12)
C8—C7—C6	119.08 (12)	O1—C16—C15	115.28 (11)
C8—C7—H7	120.5	C17—C16—C15	119.65 (12)
C6—C7—H7	120.5	C16—C17—C18	119.03 (12)
C7—C8—C9	120.94 (12)	C16—C17—H17	120.5
C7—C8—H8	119.5	C18—C17—H17	120.5
C9—C8—H8	119.5	C13—C18—C17	121.91 (11)
C10—C9—C8	121.49 (12)	C13—C18—H18	119.0
C10—C9—H9	119.3	C17—C18—H18	119.0
C8—C9—H9	119.3	O1—C19—H19A	109.5
C9—C10—C11	117.58 (12)	O1—C19—H19B	109.5
C9—C10—H10	121.2	H19A—C19—H19B	109.5
C11—C10—H10	121.2	O1—C19—H19C	109.5
N2—C11—C10	130.50 (11)	H19A—C19—H19C	109.5
N2—C11—C6	107.71 (11)	H19B—C19—H19C	109.5
			
C11—N2—C4—C5	0.24 (14)	C5—C6—C11—C10	179.05 (11)
C11—N2—C4—C12	−176.34 (11)	C13—N3—C12—C4	178.13 (11)
N2—C4—C5—C6	−0.69 (14)	C5—C4—C12—N3	−171.94 (13)
C12—C4—C5—C6	175.63 (12)	N2—C4—C12—N3	3.94 (19)
C4—C5—C6—C7	−179.42 (14)	C12—N3—C13—C18	165.19 (12)
C4—C5—C6—C11	0.87 (14)	C12—N3—C13—C14	−15.23 (19)
C11—C6—C7—C8	0.81 (18)	C18—C13—C14—C15	−3.23 (19)
C5—C6—C7—C8	−178.88 (13)	N3—C13—C14—C15	177.21 (12)
C6—C7—C8—C9	−0.08 (19)	C13—C14—C15—C16	0.4 (2)
C7—C8—C9—C10	−0.8 (2)	C19—O1—C16—C17	3.66 (18)
C8—C9—C10—C11	0.89 (19)	C19—O1—C16—C15	−176.29 (12)
C4—N2—C11—C10	−179.44 (13)	C14—C15—C16—O1	−177.25 (11)
C4—N2—C11—C6	0.32 (14)	C14—C15—C16—C17	2.80 (19)
C9—C10—C11—N2	179.59 (12)	O1—C16—C17—C18	177.07 (11)
C9—C10—C11—C6	−0.14 (19)	C15—C16—C17—C18	−2.98 (18)
C7—C6—C11—N2	179.51 (11)	C14—C13—C18—C17	3.04 (19)
C5—C6—C11—N2	−0.73 (13)	N3—C13—C18—C17	−177.36 (11)
C7—C6—C11—C10	−0.71 (19)	C16—C17—C18—C13	0.05 (19)

### Hydrogen-bond geometry (Å, º)

*Cg*1 and *Cg*2 are the centroids of the N2/C4–C6/C11 and C6–C11 rings, respectively.

**Table:**

*D*—H···*A*	*D*—H	H···*A*	*D*···*A*	*D*—H···*A*
C10—H10···O1^i^	0.93	2.58	3.2479 (16)	129
C14—H14···*Cg*1^ii^	0.93	2.81	3.6006 (14)	143
C15—H15···*Cg*2^ii^	0.93	2.79	3.5153 (14)	136
C17—H17···*Cg*1^i^	0.93	2.89	3.5718 (14)	131
C19—H19*C*···*Cg*2^i^	0.96	2.97	3.7716 (16)	142

Symmetry codes: (i) −*x*+1/2, *y*+1/2, −*z*+3/2; (ii) −*x*+3/2, *y*−1/2, −*z*+3/2.
